# Supplementary material for: Outdoor physical activity, residential green spaces and the risk of dementia in the UK Biobank cohort
Source: Commun Med (Lond). 2025 Sep 17;5:389. doi: 10.1038/s43856-025-01130-z (PMC12443963; doi:10.1038/s43856-025-01130-z)
Supplement: Supplementary file 2 — Supplementary Tables and Figures [file 43856_2025_1130_MOESM2_ESM.pdf]

SUPPLEMENTARY INFORMATION

Supplementary Table 1: Hazard ratio and 95% confidence interval of all-cause dementia in relation to outdoor activities by green space 2

Supplementary Table 2: Baseline characteristics of study participants in the analytical sample used for the neuroimaging analysis (aim 3) 3

Supplementary Table 3: Association between outdoor activities and incident dementia by other residential factors relevant to green spaces 4

Supplementary Table 4: Association between outdoor activity, green spaces (1000 m buffer), and neuroimaging markers at the first neuroimaging visit 5

Supplementary Table 5: Association between outdoor activities and dementia, excluding cases of incident dementia within 10 years of the baseline visit 6

Supplementary Figure 1: Association between outdoor activities and green space percentage (1000-meter buffer) 7

Supplementary Figure 2: Association between outdoor activities and incident dementia by residential green space (1000-meter buffer zone) 8

Supplementary Table 1: Hazard ratio and 95% confidence interval of all-cause dementia in relation to outdoor activities by green space

	More accessible green space			Less accessible green space	
	Total/Cases	HR, 95% CI		Total/Cases	HR, 95% CI
**Sum score of outdoor activities**					
Q1	8688/335	1.00 (1.00, 1.00)		32531/1430	1.00 (1.00, 1.00)
Q2	9870/336	0.89 (0.77, 1.04)		31735/1289	0.95 (0.88, 1.03)
Q3	10708/356	0.86 (0.74, 1.00)		31186/1160	0.88 (0.82, 0.96)
Q4	12446/358	0.73 (0.63, 0.85)		29687/1120	0.86 (0.79, 0.93)
**Walking**					
Q1	7656/236	1.00 (1.00, 1.00)		24168/886	1.00 (1.00, 1.00)
Q2	8049/238	0.96 (0.80, 1.14)		24112/848	0.96 (0.87, 1.05)
Q3	8295/269	1.03 (0.87, 1.23)		23736/920	1.05 (0.96, 1.15)
Q4	8881/296	1.03 (0.86, 1.22)		23312/1032	1.15 (1.05, 1.26)
**Light DIY**					
Q1	5005/177	1.00 (1.00, 1.00)		16747/640	1.00 (1.00, 1.00)
Q2	5287/159	0.87 (0.71, 1.08)		16506/582	0.93 (0.83, 1.04)
Q3	5849/157	0.78 (0.63, 0.97)		16226/540	0.88 (0.79, 0.99)
Q4	6848/190	0.77 (0.63, 0.95)		15406/562	0.93 (0.83, 1.04)
**Heavy DIY**					
Q1	4676/155	1.00 (1.00, 1.00)		13474/496	1.00 (1.00, 1.00)
Q2	5076/160	0.96 (0.77, 1.19)		13250/447	0.95 (0.83, 1.08)
Q3	5444/154	0.85 (0.69, 1.07)		12922/457	0.99 (0.88, 1.13)
Q4	5936/168	0.83 (0.67, 1.04)		12447/488	1.05 (0.92, 1.19)
**Other Excercise**					
Q1	4806/147	1.00 (1.00, 1.00)		14382/498	1.00 (1.00, 1.00)
Q2	4830/140	0.90 (0.72, 1.14)		14296/426	0.86 (0.75, 0.98)
Q3	4848/126	0.82 (0.65, 1.05)		14292/460	0.93 (0.82, 1.06)
Q4	4875/147	0.89 (0.71, 1.12)		13972/480	0.92 (0.81, 1.04)

This table provides the detailed results shown in Figure 4. All models were adjusted for individual-level factors (age at neuroimaging visit, sex, education, cardiovascular health, somatic health, and residential length) and residential-level factors (neighborhood deprivation index, air pollution, and noise level).
HR=harzard ratio, CI=confidence interval, DIY= do it yourself.

Supplementary Table 2: Baseline characteristics of study participants in the analytical sample used for the neuroimaging analysis (aim 3)

Characteristics	Total
	(N=36 854)
Age at baseline (years), mean (SD)	55.0 (7.5)
Age at neuroimaging visit (years), mean (SD)	64.4 (7.6)
Sex, n (%)	
Female	19 446 (52.8)
Male	17 408 (47.2)
Education, n (%)	
Without a college or university degree	16 808 (45.6)
College or university degree or higher	17 181 (46.6)
Unknown	2865 (7.8)
Smoking Status, n (%)	
Current	2184 (5.9)
Never	22 439 (60.9)
Previous	12 146 (33.0)
Unknown	85 (0.2)
BMI categories, n (%)	
Underweight (<18.5)	153 (0.4)
Normal (18.5≤BMI<25)	14 548 (39.5)
Overweight (25≤BMI<30)	15 764 (42.8)
Obese (≥30)	6342 (17.2)
Data missing	47 (0.1)
Diabetes, n (%)	1046 (2.8)
High Cholesterol, n (%)	14 705 (39.9)
Hypertension, n (%)	16 188 (43.9)
Outdoor activity (100 MET-min/week), median (IQR)	11.7 (5.5 – 21.3)
Outdoor activity, categories, n (%)	
First quartile (<435.0)	9214 (25.0)
Second quartile (435.0≤GE<956.3)	9214 (25.0)
Third quartile (956.3≤GE<1808.8)	9213 (25.0)
Fourth quartile (≥1808.8)	9213 (25.0)
GS (300m buffer) in %, mean (SD)	37.8 (24.2)
GS quartiles 300 m buffer, n (%)	
First quartile (<18.5)	9214 (25.0)
Second quartile (18.5≤GS<32.6)	9214 (25.0)
Third quartile (32.6≤GS<53.2)	9213 (25.0)
Fourth quartile (≥53.2)	9213 (25.0)
GS (1000m buffer) in %, mean (SD)	37.8 (24.1)
IMD, mean (SD)	15.2 (11.9)
Air Pollution (PM_{10}), mean (SD)	21.7 (2.7)
Noise Pollution (LAeq), mean (SD)	51.2 (4.2)
Residential length (year), median (IQR)	15.0 (8.0 – 24.0)

Abbreviations. SD = Standard Deviation; BMI = Body Mass Index; IQR = Interquartile Range; GS = Green Space; IMD = Index of Multiple Deprivation; PM = Particulate Matter, LAeq = A-weighted equivalent sound level

Supplementary Table 3: Association between outdoor activities and incident dementia by other residential factors relevant to green spaces

	Participants	Events	Hazard ratio	95% CI	P-value
Green Space, GS (1000m Buffer)					
Outdoor activities × high GS	41 712	1385	0.94	0.89, 1.00	0.043
Stratification Analysis					
Low GS	125 139	4837	0.94	0.92, 0.97	<0.001
High GS	41 712	1385	0.88	0.84, 0.93	<0.001
Nature Environment (300m Buffer)					
Outdoor activities × high Nature Environment	46 656	1619	0.95	0.90, 1.00	0.042
Stratification Analysis					
Low Nature Environment	139 968	5566	0.95	0.92, 0.97	<0.001
High Nature Environment	46 656	1619	0.90	0.85, 0.94	<0.001
Blue Space (1000m Buffer)					
Outdoor activities × high Blue Space	41 712	1586	1.01	0.95, 1.06	0.87
Stratification Analysis					
Low Blue Space	125 139	4798	0.92	0.90, 0.95	<0.001
High Blue Space	41 712	1586	0.94	0.89, 0.99	0.013
Domestic Garden Percentage (300m Buffer)					
Outdoor activities × high Domestic Garden Percentage	41 712	1526	1.00	0.94, 1.06	0.95
Stratification Analysis					
Low Domestic Garden Percentage	125 139	4858	0.93	0.90, 0.96	<0.001
High Domestic Garden Percentage	41 712	1526	0.92	0.88, 0.97	0.002

Model was adjusted for individual-level factors (age at neuroimaging visit, sex, education, diabetes, cholesterol, hypertension, residential length), residential-level factors (index of multiple deprivations of neighborhood, air pollution, and noise level).
GS=green space, CI=confidence interval.
Outdoor activity was treated as a continuous variable using log-transformed 100 MET-min/week.

Supplementary Table 4: Association between outdoor activity, green spaces (1000 m buffer), and neuroimaging markers at the first neuroimaging visit

		Less accessible Green Space (1000 m buffer) (n=27 641)			More accessible Green Space (1000 m buffer) (n=9213)	
Hippocampus		β coefficients (95% CI)	β coefficients (95% CI)		β coefficients (95% CI)	β coefficients (95% CI)
Outdoor activity, categories		Model 1[a]	Model 2[a]		Model 1[a]	Model 2[a]
First quartile		Ref.	Ref.		Ref.	Ref.
Second quartile		-7.27 (-27.93, 13.39)	-5.80 (-26.45, 14.85)		22.29 (-16.76, 61.34)	22.74 (-16.28, 61.77)
Third quartile		17.56 (-3.34, 38.46)	19.74 (-1.13, 40.62)		31.51 (-7.03, 70.05)	31.85 (-6.66, 70.36)
Fourth quartile		6.49 (-14.68, 27.66)	9.27 (-11.89, 30.42)		27.55 (-10.32, 65.43)	27.04 (-10.84, 64.93)
Outdoor activity , continuous[b]		4.97 (-3.31, 13.24)	6.17 (-2.09, 14.44)		11.41 (-3.47, 26.30)	11.09 (-3.80, 25.99)
Total gray matter		β coefficients (95% CI)	β coefficients (95% CI)		β coefficients (95% CI)	β coefficients (95% CI)
Outdoor activity, categories		Model 1[a]	Model 2[a]		Model 1[a]	Model 2[a]
First quartile		Ref.	Ref.		Ref.	Ref.
Second quartile		718.30 (-449.01, 1885.61)	636.15 (-530.02, 1802.33)		-297.89 (-2452.19, 1856.42)	-381.60 (-2535.25, 1772.06)
Third quartile		1224.88 (43.81, 2405.95)*	1106.10 (-73.00, 2285.20)		-152.50 (-2278.66, 1973.67)	-253.00 (-2378.20, 1872.20)
Fourth quartile		1995.15 (798.79, 3191.95)**	1806.29 (611.54, 3001.04)**		752.26 (-1337.06, 2841.58)	699.09 (-1391.57, 2789.76)
Outdoor activity, continuous[b]		842.83 (375.29, 1310.38)***	765.69 (298.98, 1232.39)**		484.86 (-336.29, 1306.02)	464.09 (-357.73, 1285.92)
WMH		β coefficients (95% CI)	β coefficients (95% CI)		β coefficients (95% CI)	β coefficients (95% CI)
Outdoor activity, categories		Model 1[a]	Model 2[a]		Model 1[a]	Model 2[a]
First quartile		Ref.	Ref.		Ref.	Ref.
Second quartile		-0.02 (-0.05, 0.00)	-0.03 (-0.05, 0.00)		-0.01 (-0.07, 0.04)	-0.02 (-0.07, 0.04)
Third quartile		-0.05 (-0.08, -0.02)***	-0.05 (-0.08, -0.03)***		-0.02 (-0.08, 0.03)	-0.03 (-0.08, 0.02)
Fourth quartile		-0.05 (-0.08, -0.03)***	-0.06 (-0.09, -0.03)***		-0.04 (-0.09, 0.01)	-0.04 (-0.09, 0.01)
Outdoor activity, continuous[b]		-0.03 (-0.04, -0,02)***	-0.03(-0.04, -0,02)***		-0.02 (-0.04, 0.00)	-0.02 (-0.04, 0.00)

[a]Model 1 was adjusted for individual-level factors (age at neuroimaging visit, sex, education, body mass index, diabetes, cholesterol, hypertension, residential length) and index of multiple deprivations.
[a]Model 2 was adjusted for individual-level factors (age at neuroimaging visit, sex, education, body mass index, diabetes, cholesterol, hypertension, residential length), air pollution, and noise level. WMH=white matter hyperintensity; CI=confidence interval. [b]Outdoor activity was treated as a continuous variable using log-transformed 100 MET-min/week. *P<0.05; **P<0.01; ***P<0.001.

Supplementary Table 5: Association between outdoor activities and dementia, excluding cases of incident dementia within 10 years of the baseline visit

	Hazard ratios (95% CI)[a]		Hazard ratios (95% CI)[a]		Hazard ratios (95% CI)[a]
	Total sample (n=184 628)		Less accessible green space (300 m buffer) (n=122 951)		More accessible green space (300 m buffer) (n=41 149)
All-cause dementia					
Outdoor activity, categories					
First quartile	Ref.		Ref.		Ref.
Second quartile	1.00 (0.92, 1.08)		0.99 (0.89, 1.09)		0.99 (0.81, 1.21)
Third quartile	0.92 (0.84, 1.00)		0.89 (0.80, 0.99)*		0.98 (0.81, 1.20)
Fourth quartile	0.91 (0.84, 1.00)*		0.93 (0.83, 1.03)		0.82 (0.67, 1.00)
Outdoor activity, continuous[b]	0.96 (0.93, 0.99)**		0.97 (0.93, 1.01)		0.91 (0.85, 0.98)**
Alzheimer's Disease					
Outdoor activity, categories					
First quartile	Ref.		Ref.		Ref.
Second quartile	1.10 (0.97, 1.24)		1.09 (0.96, 1.28)		1.08 (0.79, 1.45)
Third quartile	1.01 (0.90, 1.16)		1.00 (0.86, 1.17)		1.08 (0.80, 1.47)
Fourth quartile	0.98 (0.86, 1.12)		1.03 (0.83, 1.14)		0.95 (0.70, 1.29)
Outdoor activity, continuous[b]	0.98 (0.94, 1.03)		0.98 (0.93, 1.03)		0.96 (0.86, 1.06)
Vascular dementia					
Outdoor activity, categories					
First quartile	Ref.		Ref.		Ref.
Second quartile	0.83 (0.68, 1.00)*		0.74 (0.58, 0.93)*		0.88 (0.55, 1.40)
Third quartile	0.80 (0.66, 0.97)*		0.74 (0.58, 0.94)*		1.05 (0.68, 1.64)
Fourth quartile	0.82 (0.68, 0.99)*		0.81 (0.64, 1.02)		0.84 (0.53, 1.31)
Outdoor activity, continuous[b]	0.92 (0.86, 0.99)*		0.92 (0.85, 1.00)		0.95 (0.82, 1.12)
Frontotemporal dementia					
Outdoor activity, categories					
First quartile	Ref.		Ref.		Ref.
Second quartile	0.74 (0.38, 1.44)		0.85 (0.35, 2.06)		0.50 (0.12, 2.11)
Third quartile	0.58 (0.28, 1.18)		0.75 (0.30, 1.87)		0.14 (0.02, 1.17)
Fourth quartile	0.71 (0.37, 1.40)		1.05 (0.44, 2.44)		0.32 (0.07, 1.38)
Outdoor activity, continuous[b]	0.80 (0.62, 1.02)		0.94 (0.68, 1.29)		0.52 (0.29, 0.92)*

[a]Model was adjusted for individual-level factors (age at neuroimaging visit, sex, education, body mass index, diabetes, cholesterol, hypertension, residential length), index of multiple deprivations, air pollution, and noise level.
CI=confidence interval.
[b]Sum of outdoor activities was treated as a continuous variable using log-transformed 100 MET-min/week.

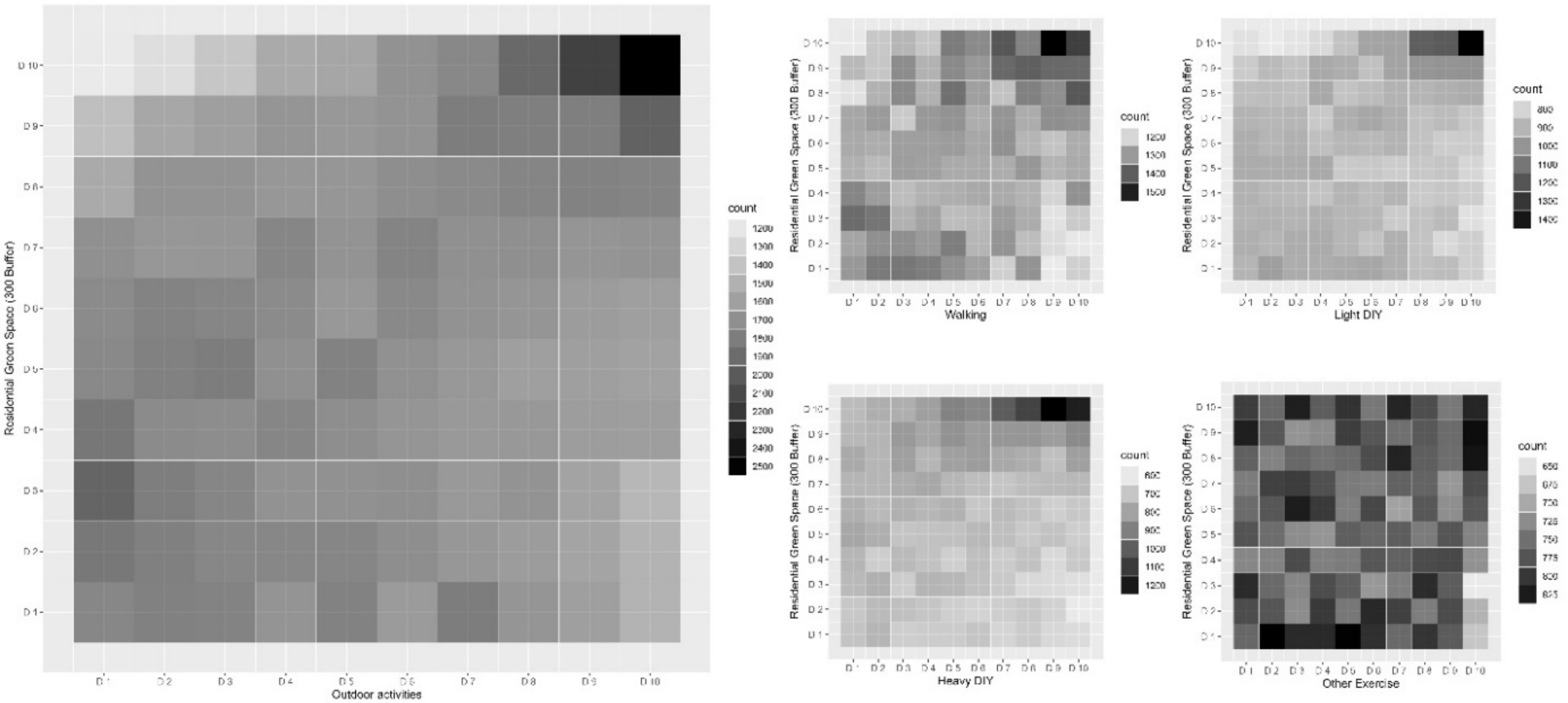

Supplementary Figure 1: Association between outdoor activities and green space percentage (1000-meter buffer)

Notes. This density plot illustrates the distribution of individuals based on their levels of outdoor activity and the percentage of green space within a 1000-meter buffer around their residence. Both the outdoor activity variable (measured in Metabolic Equivalent of Task [MET]-minutes) and the green space variable (measured as a percentage) were categorized into deciles, represented on the x-axis and y-axis, respectively. The plot displays density as the frequency of individuals within each grid cell, with darker colors indicating a higher concentration of individuals corresponding to specific deciles of outdoor activity and green space. From this plot, we can see that individuals living in areas with higher deciles of green space are more likely to engage in higher deciles of outdoor activity, such as walking, light DIY, and heavy DIY.

Abbreviations. Light DIY=light do-it-yourself activities; heavy DIY=heavy do-it-yourself activities

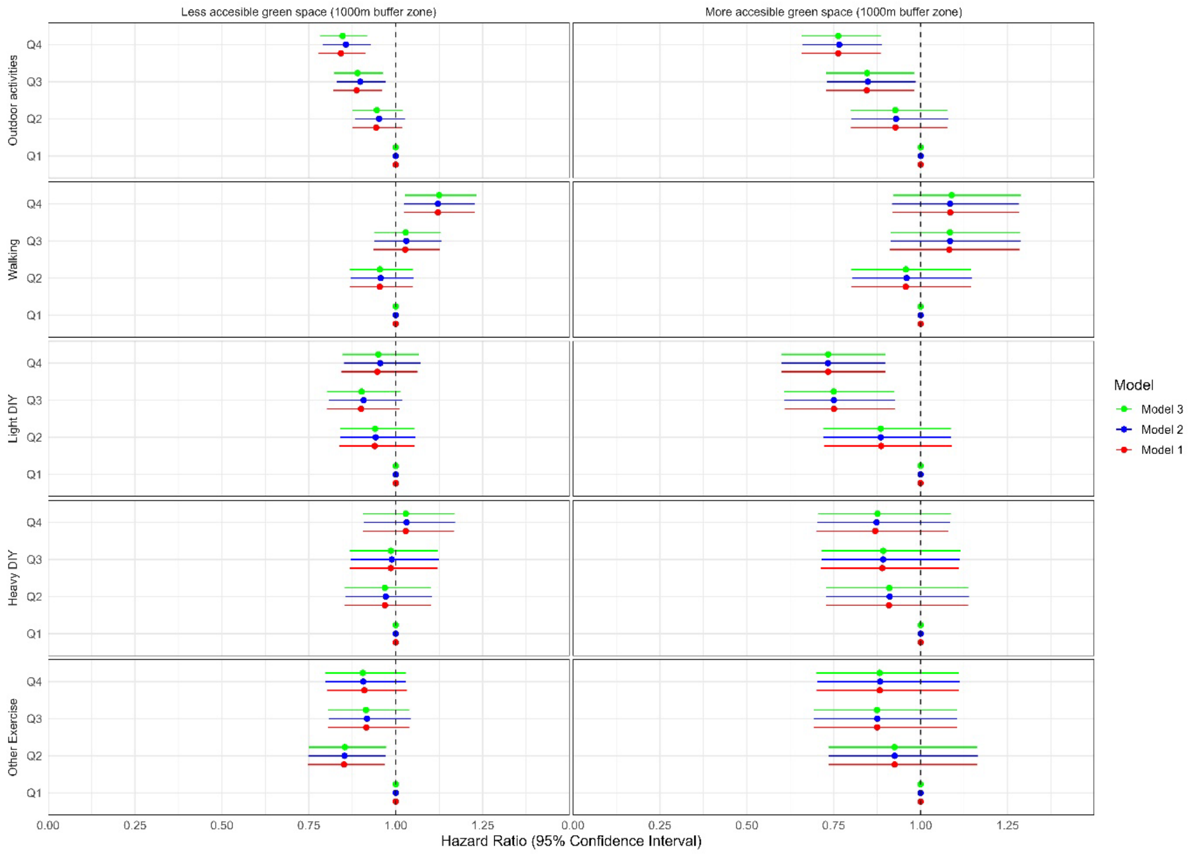

Supplementary Figure 2: Association between outdoor activities and incident dementia by residential green space (1000-meter buffer zone)

Model 1 was adjusted for individual-level factors: age, sex, education, smoking, diabetes, cholesterol, hypertension
Model 2 was Model 1+ neighborhood social index
Model 3 was Model 1 + residential air pollution and noise level
More accessible green space was categorized as the top quantile of residential green space at 1000 m buffer zone (≥62.36 %), and less accessible green space was identified with the remaining quartiles (<62.36%).
Error bars represent 95% confidence intervals.
Abbreviations. Light DIY=light do-it-yourself activities; heavy DIY=heavy do-it-yourself activities.
