# Supplementary material for: Outdoor physical activity, residential green spaces and the risk of dementia in the UK Biobank cohort
Source: Commun Med (Lond). 2025 Sep 17;5:389. doi: 10.1038/s43856-025-01130-z (PMC12443963; doi:10.1038/s43856-025-01130-z)
Supplement: Supplementary file 3 — description of additional supplementary files [file 43856_2025_1130_MOESM3_ESM.pdf]

Description of Additional Supplementary Files

File name: Supplementary Data 1

Description: Assessments of outdoor activities and dementia

File name: Supplementary Data 2

Description: Figure 2 source data

File name: Supplementary Data 3

Description: Figure 3 source data

File name: Supplementary Data 4

Description: Figure 4 source data
